# Salivary Biomarkers in Alzheimer’s Disease: Emerging Diagnostic Tools and Their Association with Periodontal Disease

**DOI:** 10.3390/ijms27135888

**Published:** 2026-06-30

**Authors:** Agata Świątek, Aida Kusiak, Adrian Maj

**Affiliations:** Department of Periodontology, Medical University of Gdańsk, 80-210 Gdansk, Poland

**Keywords:** Alzheimer’s disease, salivary biomarkers, saliva, periodontitis, oral–brain axis, amyloid-β, tau protein, lactoferrin, microRNA, neurodegeneration

## Abstract

Alzheimer’s disease (AD) is the most common neurodegenerative disorder and a leading cause of dementia worldwide. Current diagnostic methods, including cerebrospinal fluid analysis and neuroimaging, are often invasive, expensive, and not suitable for large-scale screening. Therefore, increasing attention has been directed toward the identification of non-invasive biomarkers. Saliva has emerged as a promising diagnostic biofluid containing proteins, metabolites, inflammatory mediators, exosomes, and nucleic acids potentially associated with neurodegenerative processes. This review aimed to summarize current evidence regarding salivary biomarkers in Alzheimer’s disease and to discuss their diagnostic potential, limitations, and association with periodontal disease within the framework of the oral–brain axis. A literature search was conducted using PubMed, Scopus, and Google Scholar databases for studies published between 2018 and 2026. Relevant English-language articles focusing on salivary biomarkers, Alzheimer’s disease, periodontitis, and oral–brain axis interactions were included. Current evidence suggests that salivary biomarkers such as amyloid-beta, tau protein, lactoferrin, exosomes, oxidative stress markers, metabolites, and nucleic acid-based biomarkers may reflect the pathological mechanisms associated with Alzheimer’s disease. In addition, increasing evidence supports a relationship between chronic periodontal inflammation, oral pathogens, and neurodegenerative processes. However, substantial heterogeneity among studies, methodological variability, and a lack of standardized protocols currently limit the reproducibility and clinical applicability of saliva-based diagnostics. Salivary biomarkers represent a promising non-invasive approach for the early detection and monitoring of Alzheimer’s disease. Nevertheless, further large-scale, longitudinal, and standardized studies are necessary to validate their diagnostic utility and support their implementation in routine clinical practice.

## 1. Introduction

Neurodegenerative diseases are among the most common disorders affecting the elderly population, with Alzheimer’s disease (AD) representing the leading cause of dementia worldwide [1]. AD is a progressive and irreversible neurodegenerative disorder characterized by gradual cognitive decline, memory impairment, and behavioral disturbances, ultimately leading to severe disability and death [2]. The disease was first described in 1906 by the German psychiatrist and neuropathologist Alois Alzheimer, who identified amyloid plaques and extensive neuronal degeneration in the brain tissue of a patient presenting with memory loss and personality changes [1,2].

Currently, more than 55 million people worldwide are affected by Alzheimer’s disease, and the prevalence is expected to increase substantially with population aging [3]. The greatest rise in the number of affected individuals is predicted in developing countries, where demographic changes and increased life expectancy contribute to the growing burden of neurodegenerative diseases [4]. Importantly, the pathological changes associated with AD may develop many years before the onset of clinical symptoms, highlighting the urgent need for reliable methods enabling early diagnosis and disease monitoring.

The pathogenesis of Alzheimer’s disease is complex and multifactorial. The main pathological hallmarks of AD include extracellular deposition of amyloid-β (Aβ) peptides in the form of senile plaques, intracellular accumulation of hyperphosphorylated tau protein forming neurofibrillary tangles (NFTs), chronic neuroinflammation, oxidative stress, and progressive neuronal loss [5,6]. In recent years, increasing attention has been directed toward the identification of biomarkers capable of reflecting these pathological processes and supporting early diagnosis.

Currently used diagnostic methods for Alzheimer’s disease, including cerebrospinal fluid (CSF) analysis and neuroimaging techniques, may provide valuable diagnostic information; however, they are often invasive, expensive, and not easily applicable in routine large-scale screening. Therefore, there is growing interest in alternative, non-invasive diagnostic approaches. Saliva has emerged as a promising diagnostic biofluid due to its simple, painless, and cost-effective collection. Importantly, saliva contains a wide range of biomolecules, including proteins, metabolites, inflammatory mediators, exosomes, and nucleic acids, which may reflect both systemic and neurodegenerative changes.

Moreover, increasing evidence supports the existence of an oral–brain axis linking oral health, chronic inflammation, and neurodegenerative diseases. In particular, periodontal disease and periodontal pathogens such as *Porphyromonas gingivalis* have been implicated in mechanisms associated with neuroinflammation and Alzheimer’s disease progression. These observations further support the potential relevance of salivary biomarkers in AD diagnostics.

Unlike previous reviews that primarily focused on individual salivary biomarkers or the diagnostic aspects of Alzheimer’s disease, the present review provides an integrated overview of protein-based, metabolomic, exosomal, and nucleic acid–based salivary biomarkers while simultaneously examining their relationship with periodontal disease and the oral–brain axis. In addition, this review critically evaluates the current evidence, highlights methodological limitations, and discusses challenges that must be addressed before saliva-based biomarkers can be translated into routine clinical practice.

The aim of this review is to summarize current evidence regarding salivary biomarkers in Alzheimer’s disease and to discuss their potential diagnostic value, limitations, and association with periodontal disease within the framework of the oral–brain axis.

## 2. Literature Search Strategy

A comprehensive literature search was conducted using PubMed, Scopus, Web of Science and Google Scholar databases for studies published between March 2018 and April 2026. The search strategy combined terms related to “Alzheimer’s disease”, “saliva”, “salivary biomarkers”, “periodontitis”, “oral–brain axis”, “tau protein”, “amyloid-β”, “lactoferrin”, and “microRNA”. Studies were included if they were published in English and available in full text; investigated salivary biomarkers associated with Alzheimer’s disease; evaluated the relationship between periodontal disease, oral pathogens and Alzheimer’s disease; reported findings form clinically diagnosed Alzheimer’s disease patients, mild cognitive impairment subjects or cognitively healthy controls.

Studies were excluded if they were conference abstracts, editorials, letters, case reports, duplicate publications, non-English articles, or studies lacking sufficient methodological information or relevance to the review topic. Particular attention was paid to differences in study populations, including clinically confirmed Alzheimer’s disease cohorts and mixed memory–clinic populations, as these factors may influence biomarker performance and study comparability.

The selection process involved title and abstract screening followed by full-text assessment of potentially relevant articles. Priority was given to human clinical studies whenever available, while findings from animal and in vitro studies were included primarily to support biological mechanisms underlying the oral–brain axis.

To provide a concise overview of the current clinical evidence, Table 1 summarizes representative human studies investigating salivary biomarkers in Alzheimer’s disease, including study design, investigated biomarkers, principal findings, and their potential clinical relevance.

## 3. Current Evidence on Salivary Biomarkers in Alzheimer’s Disease

### 3.1. Pathophysiology of Alzheimer’s Disease

Alzheimer’s disease (AD) is a progressive neurodegenerative disorder characterized by a complex interplay of molecular and cellular mechanisms leading to neuronal dysfunction and loss. The hallmark pathological features of AD include extracellular deposition of amyloid-β (Aβ) peptides in the form of senile plaques, intracellular accumulation of hyperphosphorylated tau protein forming neurofibrillary tangles (NFTs), and chronic neuroinflammation.

The amyloid cascade hypothesis remains one of the central theories explaining AD pathogenesis. Aβ peptides are generated through the sequential proteolytic cleavage of the amyloid precursor protein (APP) by β-secretase and γ-secretase. Among these, Aβ42 is particularly prone to aggregation and is considered the most neurotoxic form. The accumulation of Aβ leads to the formation of oligomers and fibrils, which disrupt synaptic function, induce oxidative stress, and trigger neuroinflammatory responses. Importantly, Aβ-related pathology may begin years before the onset of clinical symptoms.

Tau protein, a microtubule-associated protein, plays a critical role in stabilizing microtubules and maintaining neuronal structure. In AD, tau undergoes abnormal hyperphosphorylation, which reduces its affinity for microtubules and promotes its aggregation into paired helical filaments. These aggregates form neurofibrillary tangles within neurons, leading to cytoskeletal disruption, impaired axonal transport, and ultimately neuronal death. The extent of tau pathology has been shown to correlate more closely with cognitive decline than amyloid burden.

Neuroinflammation is another key component of AD pathophysiology. The activation of microglia and astrocytes in response to Aβ deposition results in the release of pro-inflammatory cytokines, chemokines, and reactive oxygen species. While initially protective, chronic activation of the immune response contributes to neuronal damage and disease progression. Oxidative stress further exacerbates this process by damaging proteins, lipids, and nucleic acids, thereby impairing neuronal function.

Emerging evidence also highlights the role of vascular dysfunction, mitochondrial impairment, and the dysregulation of calcium homeostasis in AD. The disruption of the blood–brain barrier may facilitate the accumulation of neurotoxic substances and impair clearance of Aβ. Additionally, disturbances in calcium signaling contribute to synaptic dysfunction and neuronal apoptosis.

Overall, the pathophysiology of Alzheimer’s disease is multifactorial, involving the interaction of amyloid deposition, tau pathology, neuroinflammation, and systemic factors. Understanding these mechanisms is essential for the development of early diagnostic strategies and novel therapeutic approaches.

### 3.2. Risk Factors of Alzheimer’s Disease

Alzheimer’s disease is a multifactorial neurodegenerative disorder influenced by both non-modifiable and modifiable risk factors. Age remains the most significant determinant, while genetic predisposition, particularly the presence of the APOE ε4 allele, further increases susceptibility. In addition, a growing body of evidence highlights the role of modifiable factors, including cardiovascular conditions, lifestyle habits, and chronic inflammation. Emerging data also suggest a potential link between periodontal disease and Alzheimer’s disease, supporting the concept of an oral–brain axis [17].

Alzheimer’s disease is influenced by a broad spectrum of non-modifiable and modifiable risk factors, including genetic susceptibility, cardiovascular and metabolic disorders, lifestyle habits, and chronic inflammatory conditions. Recent evidence has also highlighted the potential contribution of periodontal disease and oral pathogens to neurodegenerative processes within the framework of the oral–brain axis. The main risk factors currently associated with Alzheimer’s disease are summarized in Table 2.

### 3.3. Periodontitis and Alzheimer’s Disease

Periodontal disease or periodontitis is a chronic disease with bacterial etiology that leads to a gradual loss of the support structure of the tooth, such as the gingiva, periodontal ligament, cementum and alveolar bone. It has been assessed that periodontitis derives from a complex interaction between the host defence mechanisms and the bacterial compounds contained in the biofilm [20]. The mechanisms of damage the supporting structures operate on two fronts: on the one hand the damage is caused by direct action of the bacteria and their leukotoxins and enzymes which destroy the defense cells and the connective tissue. On the other hand there is an intense inflammatory response triggered by the host and caused by the persistence of the plaque which leads to the gradual destruction of the tissues [19]. There are also other factors that can influence the progression and severity of periodontitis. They are as follows: genetic predisposition, lifestyle factors, general health conditions (e.g., uncontrolled diabetes mellitus), obesity, malnutrition, osteoporosis and osteopenia, psychosocial stress, hormonal changes, drug intake, haematological or neurodegenerative disorders [20].

Notably, periodontitis and Alzheimer’s disease share multiple risk factors, including aging, systemic inflammation and metabolic disturbances. This overlap has led to increasing interest in the concept of the oral–brain axis, which describes potential bidirectional interactions between oral health and neurodegeneration.

The evidence linking periodontitis and Alzheimer’s disease originates from several levels of investigation, including human observational studies, experimental animal models, and mechanistic in vitro studies. While epidemiological studies have reported associations between periodontal disease and increased risk of cognitive decline or Alzheimer’s disease, mechanistic insights regarding bacterial virulence factors, neuroinflammation, and blood–brain barrier dysfunction have been derived predominantly from animal and laboratory studies. Therefore, although the available evidence supports a biologically plausible relationship between oral health and neurodegeneration, direct causal mechanisms in humans remain incompletely understood.

One of the key mechanisms linking periodontitis to Alzheimer’s disease involves the dissemination of periodontal pathogens and their virulence factors into systemic circulation. These microbial components may reach the central nervous system directly or indirectly, contributing to neurodegenerative processes. In particular, bacterial products such as lipopolysaccharides (LPS) have been detected in the brains of patients with Alzheimer’s disease, supporting a potential role of chronic oral infection in these pathogenesis.

Emerging evidence highlights the contribution of oral bacteria, particularly periodontitis-associated pathogens like *P. gingivalis*, in the development of extra-oral inflammation and systemic conditions, including Alzheimer’s disease [21]. *P. gingivalis* is currently the best studied and a major bacterial pathobiont that emerges as part of a subgingival pathogenetic polymicrobial community during periodontitis. On the surface of *P. gingivalis,* there are many biomolecules, such as nucleic acid, LPS and surface-located proteinases, known as gingipains. They were determined in multiple regions of human Alzheimer’s brains, including cortical grey matter, the basal forebrain and hypothalamic regions [22]. These biomolecules have been detected within neurons and associated with NFTs, in the brain of Alzheimer’s patients. The cerebral loads of gingipains have been correlated with Alzheimer’s disease diagnosis [22]. Elevated serum levels of anti-*P. gingivalis* immunoglobulin G (IgG) have been associated with an increased risk of Alzheimer’s disease, higher incidence and impaired cognitive functions, including delay recall and calculation ability [23]. Human observational studies by Beydoun et al. have reported associations between periodontitis and increased risk of cognitive decline and dementia. In the last years, several animal studies demonstrated that administration of *P. gingivalis* or its virulence factors generated Alzheimer’s disease-like phenotypes including neuroinflammation, amyloid-β accumulation, tau hyperphosphorylation, neurodegeneration and cognitive impairments in small animal models [18]. Although bacterial components such as lipopolysaccharides (LPS), gingipains, and bacterial nucleic acids have been detected in the brains of patients with Alzheimer’s disease, there is currently no convincing evidence that viable oral bacteria actively colonize or proliferate within brain tissue. Neither human studies nor experimental animal models have consistently demonstrated the presence of living periodontal pathogens within the central nervous system. Therefore, the current consensus suggests that the effects of periodontal pathogens on the brain are predominantly mediated by the systemic dissemination of bacterial virulence factors rather than by direct bacterial invasion. In particular, outer membrane vesicles (OMVs) released by *P. gingivalis* can transport gingipains, LPS, nucleic acids, and other bioactive molecules through the circulation. These vesicles may disrupt blood–brain barrier integrity, promote neuroinflammation, and contribute to amyloid-β accumulation and tau pathology without requiring the presence of viable bacteria within brain tissue [18].

OMVs are spherical nanostructures released from the outer membrane of Gram-negative bacteria. Carrying a broad range of virulence factors, they play a pivotal role in bacterial growth, intercellular communication, biofilm formation, invasion and modulation of host defense. The bacteria of the red complex of periopathogens—*P. gingivalis*, *Tanarella forsythia* and *Prevotella intermedia*—have their outer membrane surface loaded with proteinaceous virulence factors [24]. 

Due to relatively small size, high abundance, and enrichment in proteinases and adhesins compared with their parental cells, the OMVs of *P. gingivalis* exhibit an increased capacity to invade host tissues and disseminate via the bloodstream to distant organs [18]. In a murine experimental model, Yoshida et al. demonstrated that *P. gingivalis* OMVs translocated into the brain after repeated injection into the abdominal cavity of mice for 12 weeks [25]. *P. gingivalis* OMVs may impair the blood–brain barrier (BBB) and gain access to the brain through multiple pathways [18]. Gingipains present on the surface of OMVs are capable of degrading key structural components of the BBB, including junctional proteins, integrins, and extracellular matrix proteins. Furthermore, OMVs have been shown to increase vascular permeability in a gingipain-dependent manner by cleaving endothelial adhesion molecules such as PECAM-1, a critical component of adherence junctions. Through these mechanisms, *P. gingivalis*-derived OMVs may promote neuroinflammation, facilitate the accumulation of neurotoxic molecules, and contribute to neuronal dysfunction observed in Alzheimer’s disease [26]. 

Gingipains may also degrade integrins, which may contribute to BBB disruption and brain haemorrhage [18]. They can degrade ECM proteins because of strong affinity to them and disturbing its function maintaining BBB integrity. 

Beyond direct BBB disruption, increased vascular permeability may facilitate the entry of circulating inflammatory mediators, immune cells, and potentially neurotoxic molecules into the central nervous system. Elevated systemic levels of pro-inflammatory cytokines such as interleukin-1β (IL-1β), interleukin-6 (IL-6), and tumor necrosis factor-α (TNF-α), commonly observed in chronic periodontitis [27], may, therefore, gain greater access to the brain parenchyma and amplify local inflammatory responses. In addition, BBB dysfunction may impair physiological clearance mechanisms involved in the removal of amyloid-β and other neurotoxic metabolites, thereby further promoting neurodegenerative processes [28].

Following their entry into the brain, OMVs and their cargo may interact with resident microglial cells through pattern recognition receptors, particularly Toll-like receptors (TLRs), including TLR2 and TLR4 [18,29,30]. The activation of these receptors triggers downstream NF-κB signaling pathways, resulting in the production of pro-inflammatory cytokines, chemokines, and reactive oxygen species. Furthermore, OMV-associated lipopolysaccharides and gingipains have been implicated in the activation of the NLRP3 inflammasome, leading to the enhanced maturation and secretion of IL-1β and IL-18. Chronic microglial activation may contribute to sustained neuroinflammation, synaptic dysfunction, amyloid-β accumulation, tau hyperphosphorylation, and progressive neuronal damage, thereby providing a mechanistic link between periodontal inflammation and Alzheimer’s disease pathology [28,30].

These observations further support the concept that OMVs may function not only as carriers of bacterial virulence factors but also as active modulators of neuroimmune signaling within the oral–brain axis.

Another interesting fact is association of pathogenesis of Alzheimer’s disease with iron, as a most abundant transition metal in the brain. Iron may influence the production of amyloid-β. It mediates hyperphosphorylation of tau and its dysfunction. It is related to the formation of NFTs. Ayton et al. suggested that iron can be an effector of neurodegeneration [31]. They found that brain iron within normal levels was strikingly associated with the cognition impairment in patients with established Alzheimer’s disease neuropathology [31].

Interestingly *P. gingivalis* is relying on host-derived haem and iron to meet its basic need for survival and growth. *P. gingivalis* developed various mechanisms to capture haem and iron from the host and to store haem on its surface. In this context the transfer of iron by *P. gingivalis* from the bloodstream to the brain may explain maldistribution of iron in Alzheimer’s disease. Importantly, during periodontitis progression, the expansion of *P. gingivalis* and the compromise of epithelial integrity promote the systemic spread of OMVs, which may contribute to a redistribution of iron from the circulation to the brain. These findings suggest that OMVs may act as a mechanistic bridge between chronic periodontitis and Alzheimer’s disease [18].

Another important pathway is chronic systemic inflammation. Periodontitis is associated with elevated levels of pro-inflammatory mediators, which may alter blood–brain barrier integrity and promote neuroinflammation. Sustained inflammatory signaling within the CNS has been shown to accelerate key pathological processes in Alzheimer’s disease, including amyloid-β accumulation and tau pathology. In addition, chronic inflammation may contribute to oxidative stress and immune dysregulation, further exacerbating neuronal damage.

Saliva represents a unique diagnostic medium linking oral and systemic health. Saliva contains a wide range of biomolecules, including proteins, metabolites and nucleic acids, which may reflect both local periodontal status and systemic pathological changes. Therefore, salivary biomarkers may provide valuable insight into the relationship between periodontal disease and neurodegenerative processes in Alzheimer’s disease.

Overall, although growing evidence supports an association between periodontitis and Alzheimer’s disease, the exact causal relationship remains to be fully established. Nevertheless current data suggest that periodontal disease may contribute to the pathogenesis and progression of Alzheimer’s disease through inflammatory, microbial and immune-mediated mechanisms. These findings support the potential role of saliva as a non-invasive tool for early detection and monitoring of Alzheimer’s disease.

Although accumulating evidence supports a potential relationship between periodontitis and Alzheimer’s disease, the current evidence should be interpreted with caution. Most mechanistic data originate from animal models and experimental studies, whereas human studies are predominantly observational and cannot establish causality. In addition, both conditions share several common risk factors, including advanced age, cardiovascular disease, diabetes, smoking, and socioeconomic determinants, which may contribute to the observed associations. Therefore, while the oral–brain axis represents a biologically plausible framework linking oral health and neurodegeneration, further prospective clinical studies are required to determine the extent to which periodontal disease directly contributes to Alzheimer’s disease pathogenesis.

**Figure 1:**
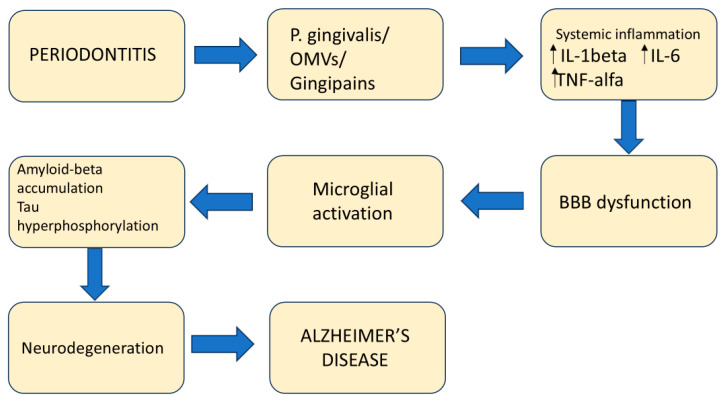
Oral–brain axis linking periodontitis and Alzheimer’s disease. Arrows indicate the proposed sequence of biological events and interactions connecting periodontal inflammation, systemic inflammatory responses, blood–brain barrier dysfunction, microglial activation, amyloid-β accumulation, tau hyperphosphorylation, neurodegeneration, and Alzheimer’s disease.

A schematic overview of the proposed oral–brain axis, illustrating the potential mechanisms linking periodontitis with neuroinflammation and Alzheimer’s disease pathology, is presented in Figure 1.

### 3.4. Diagnostic Potential of Saliva

Cerebrospinal fluid (CSF) is used as an aid in Alzheimer’s disease diagnosis and disease progression. In many cases, CSF is used to identify alterations in the level of tau, p-tau and amyloid-β. However, the sample collection is invasive and requires hospitalization [6]. Measurements are difficult to obtain and expensive. That was the reason for looking for other diagnostic approaches more affordable and non-invasive. Saliva has emerged as a promising biofluid for the identification of biomarkers in Alzheimer’s disease due to its non-invasive, cost-effective, and easily repeatable collection. Unlike cerebrospinal fluid sampling or neuroimaging techniques, saliva collection does not require specialized equipment or trained personnel, making it suitable for large-scale screening. Importantly, saliva contains a wide range of biomolecules, including proteins, metabolites, and nucleic acids, which may reflect systemic and neurodegenerative processes. The concept of the oral–brain axis further supports the potential role of saliva in Alzheimer’s disease diagnostics, suggesting that pathological changes in the central nervous system may be mirrored in the oral cavity. Despite these advantages, the clinical applicability of salivary biomarkers remains limited due to methodological variability and lack of standardization.

### 3.5. Salivary Biomarkers in Alzheimer’s Disease

Saliva is an easily accessible biofluid. Studies suggest that proteins derived from the CNS may be detected in saliva [32] and is demonstrated as rich in biomarkers for the detection of different types of brain disease, such as autism spectrum disorder, Parkinson’s disease, amyotrohic lateral sclerosis, Huntington’s disease, multiple sclerosis and Alzheimer’s disease [6].

In this part we focus on the description of the currently available data on salivary biomarkers for Alzheimer’s disease.

- Amyloid beta

Amyloid-β (Aβ) is a peptide generated through the proteolytic cleavage of amyloid precursor protein (APP) by α-, β-, and γ-secretases [33]. Abnormal APP processing leads to the formation and aggregation of Aβ peptides, particularly Aβ42, which is considered the most neurotoxic form. These aggregates form extracellular amyloid plaques, one of the hallmark pathological features of Alzheimer’s disease [34].

**Figure 2:**
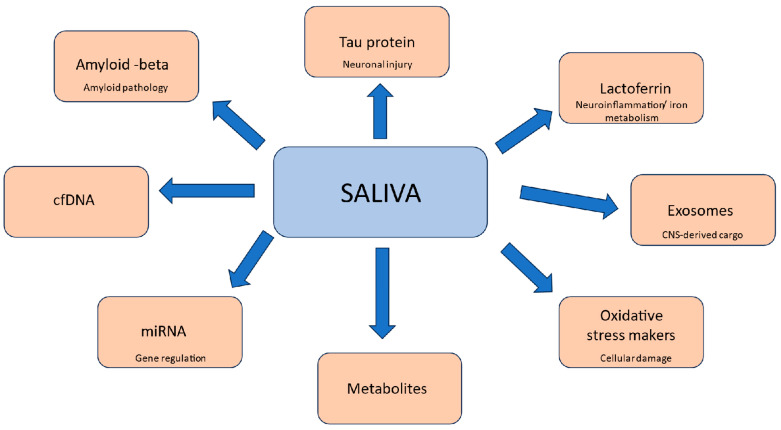
Overview of the principal salivary biomarkers investigated in Alzheimer’s disease. The figure summarizes major biomarker categories, including amyloid-β, tau protein, lactoferrin, exosomes, oxidative stress markers, metabolites, inflammatory mediators, and nucleic acid–based biomarkers, which may reflect pathological processes associated with neurodegeneration and cognitive decline.

The main categories of salivary biomarkers currently investigated in Alzheimer’s disease and their relationship to disease-associated pathological processes are summarized in Figure 2.

The accumulation of Aβ contributes to neuronal dysfunction through several mechanisms, including oxidative stress, disruption of calcium homeostasis, synaptic impairment, and neuroinflammation [35]. Due to its central role in AD pathogenesis, amyloid-β has become one of the most extensively investigated biomarkers in cerebrospinal fluid, blood, and saliva. Several studies have reported elevated salivary Aβ42 levels in patients with Alzheimer’s disease, suggesting its potential utility as a non-invasive diagnostic biomarker. However, inconsistent findings and methodological variability currently limit its clinical applicability. Furthermore, considerable differences in reported salivary Aβ concentrations between studies suggest that amyloid-β alone may not provide sufficient diagnostic accuracy and should likely be interpreted as part of a multi-biomarker approach.

- Tau protein

Tau protein promotes the assembly and stabilization of microtubules, which are essential for intracellular transport and neuronal integrity. The function of tau protein is regulated by phosphorylation [36]. Hyperphosphorylation reduces its affinity for microtubules, promoting aggregation and the formation of intracellular neurofibrillary tangles. Therefore, it loses its physiological function and leads to the destabilization of microtubules or develops toxic functions that contribute to neuronal apoptosis [37,38]. The degree of tau is related to the neuronal damage in the brain and the progression of Alzheimer’s disease [39]. Therefore, the level of p-tau and the ratio of p-tau/tau can be used for the early detection of Alzheimer’s disease and predict the rate of cognitive decline [39]. 

Evidence suggests that extracellular soluble tau is transported from the brain to peripheral tissues through arachnoid villi, the blood–cerebrospinal fluid barrier and perineural routes. As saliva reflects alterations in CSF, salivary tau levels may provide a surrogate marker of brain tau pathology in the brain [40]. 

In recent years, several studies have examined salivary p-tau levels. From them, four studies used ELISAs to detect salivary p-tau and obtained different results. Sabaei et al. [14] proved that salivary p-tau levels were higher in the Alzheimer’s disease group than in the control group. Cui et al. did not find differences in salivary p-tau between Alzheimer’s disease and controls [11]. Differences in saliva collection methods, assay sensitivity, and disease severity among study populations may partially explain these inconsistent findings. Although p-tau remains one of the most biologically relevant biomarkers of Alzheimer’s disease pathology, current salivary studies have not yet demonstrated sufficient consistency to support its routine clinical implementation.

- Lactoferrin

Lactoferrin is a glycoprotein which binds iron. It is usually present in the milk, saliva, mucosal surfaces, secondary granules of neutrophils and seminal fluid. It is primarily involved in iron metabolism, facilitating iron absorption in the human body. In addition, it plays a key role in host defence due to its antimicrobial properties. In the human brain, it exhibits several neuroprotective properties. Its anti-inflammatory and antioxidant activities contribute neuronal repair and help preserve brain integrity. In the context of Alzheimer’s disease, lactoferrin has been implicated in disease pathology, as its levels are increased in the brains of affected patients and it is found within amyloid-β plaques and intracellular neurofibrillary tangles. Importantly, lactoferrin can cross the blood–brain barrier, which separates the central and peripheral systems. Therefore, lactoferrin may represent a promising role for early detection of Alzheimer’s disease and for monitoring disease progression [40]. Zalewska et al. [10] showed that in patients with Alzheimer’s disease, an increase in beta-amyloid concentration was observed, but the amount of saliva also decreased with the level of lactoferrin. Antequera et al. [15] also reported that salivary lactoferrin levels were lower in patients with late-onset AD compared to those with early-onset AD, whereas no age-related differences were observed in control groups. Moreover, salivary lactoferrin levels showed a positive correlation with CSF Aβ42 and a negative correlation with CSF t-tau [7]. Moreover, salivary lactoferrin levels were inversely associated with cerebral amyloid deposition [8]. Studies above suggested that salivary lactoferrin can be used in detection of Alzheimer’s disease. In contrast, Gleerup et al. in 2021 [9] reported no significant differences in salivary lactoferrin levels between healthy controls and patients with Alzheimer’s disease. As well, no associations were observed between lactoferrin levels and CSF tau, CSF p-tau, or CSF Aβ42. These conflicting findings may result from differences in patient selection, disease stage, sample handling, and analytical methodology. Importantly, Gleerup et al. included a mixed memory clinic population, which may have reduced biomarker specificity compared with studies focusing exclusively on clinically confirmed AD patients.

These discrepancies are likely not attributable to a single factor, but rather to combined differences in study design, cohort selection, and analytical methodology. Studies reporting reduced salivary lactoferrin levels often included more clearly defined AD cohorts, whereas Gleerup et al. examined a mixed memory–clinic population, which may have included patients with heterogeneous cognitive disorders and different underlying pathologies. Such diagnostic heterogeneity may reduce the specificity of lactoferrin changes for Alzheimer’s disease.

Pre-analytical factors may also substantially affect lactoferrin measurements. Salivary lactoferrin concentrations can be influenced by the type of saliva collected, stimulated or unstimulated sampling, collection time, salivary flow rate, oral inflammatory status, periodontal condition, medication use, storage temperature, and freeze–thaw procedures. In addition, differences between ELISA kits, antibody specificity, detection limits, calibration standards, and intra- or inter-assay variability may contribute to inconsistent results across studies. Since lactoferrin is also an antimicrobial and neutrophil-associated protein, local oral inflammation may either mask or exaggerate disease-related alterations.

Therefore, although reduced salivary lactoferrin has been proposed as a promising non-invasive biomarker for Alzheimer’s disease, current evidence should be interpreted cautiously. Future studies should use standardized saliva collection protocols, report periodontal and oral health status, apply validated high-sensitivity assays, and compare clinically confirmed AD cohorts with well-characterized control groups.

- Salivary exosomes

Exosomes are small extracellular vehicles for various components, such as proteins, lipids and nucleic acids. They are involved in amyloid-β deposits, the formation of neurofibrillary tangles, neuroinflammation and neuronal dysfunction in Alzheimer’s disease [41]. Exosomes may have a dual role in Alzheimer’s disease by contributing to neuronal death and also helping to alleviate the pathological progression of the disease [41]. Exosomes in CNS transmit messages locally inside cells and extensively across the brain via CSF. Studies demonstrated several cells in the CNS like neurons, astrocytes, oligodendrocytes, microglia and endothelial cells may release exosomes [41]. Brain-derived exosomes can be released into saliva. Those present in CSF may be transported into the systemic circulation and ultimately reach saliva [41]. Despite their strong biological rationale and ability to transport disease-related molecules, the clinical utility of salivary exosomes remains limited by the lack of standardized isolation methods, high analytical complexity, and insufficient validation in large patient cohorts.

- Metabolites and Oxidative Stress Markers

Lipid metabolism and oxidative stress are key mechanisms in Alzheimer’s disease. The link between plasma lipid metabolites and oxidative stress in Alzheimer’s patients is poorly understood. In some studies, significant differences in lipid metabolism in patients with Alzheimer’s disease were found [41]. Lipids are involved not only in the processing of amyloid precursor protein but also in synaptogenesis, myelin formation, inflammation, oxidative stress, and other mechanisms contributing to the progression of Alzheimer’s disease pathology [41,42,43].

Oxidative stress is a well-recognized contributor to the pathogenesis of Alzheimer’s disease [44]. Oxidative stress promotes amyloid-β deposition, tau hyperphosphorylation and neuronal loss [45]. Accumulation of amyloid-β enhances oxidative stress by impairing endogenous antioxidant defence and promoting lipid peroxidation and protein oxidation, thereby creating a vicious cycle [46].

Ionescu-Tucker et al. [47] showed that mitochondrial oxidative stress is an important factor in pathogenesis of Alzheimer’s disease. Notably, there are increased levels of oxidative proteins in both the central and peripheral nervous systems.

Despite growing evidence, the association between lipid metabolites and oxidative stress in patient plasma samples remains insufficiently characterized [48], largely because most existing studies are based on animal or in vitro models. Consequently, comprehensive evaluation of lipid metabolism and oxidative stress in individuals with Alzheimer’s disease is warranted [46]. Consequently, although oxidative stress markers and lipid metabolites may provide valuable insights into disease mechanisms, their diagnostic performance remains insufficiently characterized for routine clinical application.

- Nucleic acid-based biomarkers

Nucleic acid-based biomarkers have recently emerged as promising tools for the diagnosis and monitoring of Alzheimer’s disease [40,49]. These biomarkers include DNA, messenger RNA (mRNA), microRNA (miRNA), and other non-coding RNAs, which may reflect molecular and pathological alterations associated with neurodegeneration [40,50]. Importantly, nucleic acids can be detected in saliva either in free form or enclosed within extracellular vesicles such as exosomes, which protect them from enzymatic degradation and increase their stability [50].

Among nucleic acid-based biomarkers, microRNAs (miRNAs) have attracted particular attention in Alzheimer’s disease research [40,49]. miRNAs are short non-coding RNA molecules involved in the post-transcriptional regulation of gene expression. They play an important role in numerous biological processes, including neuronal differentiation, synaptic plasticity, neuroinflammation, oxidative stress, and apoptosis. Dysregulation of specific miRNAs has been associated with amyloid-β accumulation, tau hyperphosphorylation, and progression of neurodegeneration in Alzheimer’s disease.

Several studies have demonstrated altered expression profiles of salivary miRNAs in patients with Alzheimer’s disease compared with healthy controls [13]. Specific miRNAs have been proposed as potential biomarkers due to their involvement in pathways related to inflammation, amyloid metabolism, and neuronal dysfunction [13,49]. In addition, salivary miRNAs may provide advantages as diagnostic biomarkers because of their relative stability, non-invasive accessibility, and potential to reflect central nervous system alterations [40,50].

Other nucleic acid-based biomarkers, including cell-free DNA (cfDNA), mRNA, and long non-coding RNAs (lncRNAs), are also being investigated in the context of Alzheimer’s disease [40,50]. Emerging evidence suggests that these molecules may participate in neuroinflammatory and neurodegenerative pathways and could contribute to future biomarker panels. However, current evidence remains limited, and the diagnostic value of these biomarkers has not yet been fully established.

Despite promising preliminary findings, several limitations currently restrict the clinical applicability of nucleic acid-based salivary biomarkers. Variability in saliva collection methods, sample handling, RNA isolation techniques, and analytical platforms may significantly affect study reproducibility [40]. Moreover, many studies are based on relatively small patient cohorts, and the identified biomarker profiles are often inconsistent across investigations. Therefore, further large-scale and standardized studies are necessary to validate the diagnostic utility of nucleic acid-based salivary biomarkers in Alzheimer’s disease. At present, the heterogeneity of reported biomarker signatures and the lack of reproducible findings across independent cohorts prevent their translation into clinical practice.

Table 3 summarizes the principal salivary biomarkers investigated in Alzheimer’s disease, including their biological roles, diagnostic relevance, and reported clinical findings.

A detailed summary of the principal human studies investigating salivary biomarkers in Alzheimer’s disease, including study populations, sample types, analytical techniques, and key findings, is provided in Appendix A Table A1.

## 4. Clinical Relevance, Limitations, and Future Directions

### 4.1. Clinical Relevance and Limitations

Salivary biomarkers represent a promising and non-invasive approach for the early detection and monitoring of Alzheimer’s disease (AD). Compared with cerebrospinal fluid (CSF) analysis and neuroimaging techniques, saliva collection is simple, cost-effective, painless, and easily repeatable, making it particularly attractive for large-scale screening and longitudinal monitoring. In addition, saliva sampling does not require specialized personnel or invasive procedures, which may improve patient compliance, especially among elderly individuals and cognitively impaired patients [12].

A growing body of evidence suggests that salivary biomarkers, including amyloid-β, tau protein, lactoferrin, exosomes, metabolites, oxidative stress markers, and nucleic acid–based biomarkers, may reflect pathological processes occurring in the central nervous system. Furthermore, the relationship between oral health, chronic inflammation, and neurodegeneration supports the potential diagnostic relevance of saliva within the framework of the oral–brain axis. From a clinical perspective, salivary biomarkers may contribute not only to the early detection of AD but also to disease monitoring, risk stratification, and assessment of therapeutic response [6].

Despite these promising findings, several limitations currently restrict the clinical applicability of salivary biomarkers in Alzheimer’s disease. One of the major challenges is the lack of methodological standardization [12]. Variability in saliva collection protocols, sample processing, storage conditions, and analytical techniques may significantly influence biomarker concentrations and reduce reproducibility among studies. In addition, salivary composition may be affected by numerous physiological and pathological factors, including age, circadian rhythm, medication use, hydration status, smoking, systemic diseases, and oral inflammatory conditions such as periodontitis [12].

Another important limitation is the inconsistency of available evidence. While some studies have demonstrated significant differences in salivary biomarker levels between AD patients and healthy controls, others have failed to reproduce these findings. Such discrepancies may result from differences in study populations, disease stages, sample sizes, laboratory methodologies, and biomarker detection techniques. For example, Ashton et al. [51] emphasized the promising diagnostic potential of salivary biomarkers, particularly when combined into multi-marker approaches, whereas Borelli et al. [52], in a recent systematic review, highlighted considerable heterogeneity among studies and insufficient evidence for routine clinical implementation. Similarly, Agnello et al. [16] demonstrated the feasibility of automated chemiluminescent assays for detecting AD-related biomarkers in saliva and plasma; however, they also reported variability in diagnostic performance between individual biomarkers. Moreover, many currently available studies are limited by relatively small cohorts and cross-sectional design, which reduces their generalizability [12].

A critical evaluation of currently investigated salivary biomarkers indicates that their diagnostic performance remains variable. Among the available candidates, lactoferrin and phosphorylated tau have demonstrated some of the most promising results, showing associations with established cerebrospinal fluid biomarkers and disease status in several studies. However, these findings have not been consistently replicated across independent cohorts. Similarly, salivary amyloid-β has shown potential diagnostic value, but reported concentrations vary substantially between studies, limiting its reliability. Exosomes and nucleic acid–based biomarkers are particularly attractive because they may better reflect central nervous system pathology; nevertheless, the current evidence is based on relatively small cohorts and lacks external validation. Overall, although several biomarkers demonstrate diagnostic potential, none currently exhibit sufficient accuracy, reproducibility, and standardization to support routine clinical use as a standalone test for Alzheimer’s disease.

Importantly, no single salivary biomarker has demonstrated sufficient sensitivity and specificity to serve as a standalone diagnostic tool for Alzheimer’s disease [51]. Therefore, future approaches will likely require the use of multi-biomarker panels combined with clinical, neuropsychological, imaging, and laboratory data [46]. Advances in molecular diagnostics, proteomics, metabolomics, and artificial intelligence may further improve the diagnostic value of saliva-based testing [12,16].

From a clinical implementation perspective, several challenges remain unresolved. Before salivary biomarkers can be incorporated into routine diagnostic pathways, standardized protocols for saliva collection, processing, storage, and analysis must be established. In addition, clinically relevant diagnostic cut-off values and reference ranges have not yet been defined. Most studies have been conducted in relatively small and highly selected cohorts, limiting generalizability to broader patient populations. Furthermore, regulatory approval and validation in multicenter prospective studies are required before saliva-based assays can be integrated into routine clinical practice. Consequently, salivary biomarkers should currently be considered investigational tools rather than established diagnostic tests for Alzheimer’s disease.

Not all salivary biomarkers currently under investigation are supported by the same level of evidence. Based on the available literature, salivary lactoferrin can be considered one of the most extensively studied candidates, with several independent cohorts demonstrating associations with Alzheimer’s disease pathology and established cerebrospinal fluid biomarkers. However, conflicting findings have also been reported, highlighting the need for further validation.

P-tau and amyloid-β exhibit strong biological relevance because they directly reflect core pathological hallmarks of Alzheimer’s disease. Nevertheless, salivary studies have produced inconsistent results, and their diagnostic performance remains insufficiently established.

Nucleic acid-based biomarkers, particularly microRNAs, and salivary exosomes represent emerging biomarker classes with promising preliminary findings. However, current evidence is derived from relatively small cohorts and lacks sufficient replication across independent populations.

In contrast, oxidative stress markers, inflammatory mediators, and metabolic alterations are primarily valuable for understanding disease mechanisms and systemic processes associated with neurodegeneration. Although they may contribute to multi-biomarker diagnostic panels, their specificity for Alzheimer’s disease is currently limited.

Therefore, current evidence suggests that salivary biomarkers should not be considered equally mature diagnostic candidates. Lactoferrin, amyloid-β, and p-tau currently show the greatest diagnostic potential, whereas exosomes, nucleic acid–based biomarkers, and oxidative stress markers remain largely investigational and require further clinical validation.

Overall, although salivary biomarkers show considerable clinical potential, further large-scale, longitudinal, and standardized studies are necessary before saliva can be implemented as a reliable diagnostic tool in routine clinical practice for Alzheimer’s disease.

Given the heterogeneity of the available literature and the varying stages of biomarker development, Table 4 provides an overview of the current level of evidence, independent validation status, and potential clinical applicability of the principal salivary biomarkers investigated in Alzheimer’s disease.

The available evidence is summarized in Appendix A Table A1, which highlights substantial heterogeneity in study design, cohort characteristics, sample processing protocols, and analytical methodologies across investigations.

### 4.2. Future Directions and Emerging Trends

Despite considerable progress in identifying salivary biomarkers for Alzheimer’s disease, several challenges remain before their routine clinical implementation can be achieved. Future research should focus on the standardization of saliva collection, processing, and analytical methodologies to improve reproducibility and comparability across studies. Large-scale, multicenter longitudinal studies are needed to validate the diagnostic and prognostic value of candidate biomarkers in diverse populations.

Emerging technologies, including high-throughput proteomics, metabolomics, transcriptomics, and multi-omics approaches, may facilitate the identification of novel biomarker panels with improved sensitivity and specificity. Rather than relying on a single biomarker, future diagnostic strategies will likely involve combinations of proteins, nucleic acids, metabolites, and inflammatory markers integrated into composite risk models.

Artificial intelligence (AI) and machine learning algorithms are expected to play an increasingly important role in analyzing complex biomarker datasets and identifying disease-specific patterns that may not be detectable using conventional statistical methods. These computational approaches could support early diagnosis, risk stratification, and personalized monitoring of disease progression.

Furthermore, growing evidence supporting the oral–brain axis suggests that future studies should investigate the combined assessment of salivary biomarkers and oral health parameters, particularly periodontal status, to better understand their contribution to neurodegenerative processes. The integration of salivary diagnostics with digital health technologies and point-of-care testing devices may ultimately enable rapid, non-invasive, and cost-effective screening for Alzheimer’s disease in both clinical and community settings.

Another promising area is the development of saliva-based liquid biopsy approaches for neurodegenerative diseases. Advances in molecular detection technologies may enable the identification of disease-specific proteins, extracellular vesicles, microRNAs, and cell-free nucleic acids with high analytical sensitivity. In the future, such approaches could support personalized medicine strategies by facilitating early risk assessment, individualized monitoring of disease progression, and evaluation of therapeutic responses in patients with Alzheimer’s disease.

The convergence of multi-omics technologies, liquid biopsy platforms, artificial intelligence, and personalized medicine may transform salivary diagnostics from an experimental research tool into a clinically applicable component of precision neurology.

## 5. Conclusions

Salivary biomarkers represent a promising, non-invasive approach for the early detection and monitoring of Alzheimer’s disease. Current evidence suggests that biomarkers such as amyloid-β, tau protein, lactoferrin, exosomes, oxidative stress markers, and nucleic acid-based biomarkers may reflect neurodegenerative processes occurring in the central nervous system. In addition, growing evidence supporting the oral–brain axis highlights a potential relationship between periodontitis, chronic inflammation, and Alzheimer’s disease pathogenesis.

However, despite encouraging findings, methodological variability and inconsistent study results currently limit the clinical implementation of saliva-based diagnostics. Therefore, further large-scale, longitudinal, and standardized studies are necessary to validate the diagnostic utility of salivary biomarkers and establish their role in routine clinical practice.

## Figures and Tables

**Table 1:** Characteristics of representative human studies investigating salivary biomarkers in Alzheimer’s disease.

Study	Study Design	Population	Biomarker(s) Investigated	Main Findings	Clinical Relevance
Carro et al., 2017 [7]	Case–control study	Patients with MCI, AD and healthy controls	Lactoferrin	Significantly reduced salivary lactoferrin levels in MCI and AD patients; correlated with CSF biomarkers	Suggested as a potential early non-invasive biomarker
González-Sánchez et al., 2020 [8]	Observational study	AD patients and controls	Lactoferrin	Reduced salivary lactoferrin levels were associated with cerebral amyloid pathology	Supports diagnostic value of lactoferrin
Gleerup et al., 2021 [9]	Clinical cohort study	Mixed memory clinic population	Lactoferrin	No significant differences between AD patients and controls	Highlighted limited reproducibility and need for validation
Zalewska et al., 2021 [10]	Case-control study	AD patients and healthy controls	Lactoferrin, oxidative stress markers	Reduced salivary secretion and lactoferrin levels; evidence of oxidative imbalance	Supports association between oral biomarkers and AD pathology
Cui et al., 2022 [11]	Cross-sectional study	AD patients and controls	Tau protein (p-tau)	No significant differences in salivary p-tau concentrations	Demonstrates inconsistency among tau studies
McNicholas et al., 2022 [12]	Feasibility study	Individuals with MCI, AD, and controls	Inflammatory biomarkers	Salivary inflammatory markers predicted cognitive impairment and AD status	Suggests utility of inflammatory biomarker panels
Ryu et al., 2023 [13]	Clinical biomarker study	Patients with AD	Exosomal miRNA (miR-485-3p)	Salivary exosomal miRNA levels correlated with cerebral amyloid deposition	Supports potential role of salivary miRNAs in AD diagnostics
Sabaei et al., 2023 [14]	Cross-sectional study	Early-stage AD patients and controls	Phosphorylated tau	Higher salivary p-tau levels observed in AD patients	Supports diagnostic potential of salivary tau
Antequera et al., 2024 [15]	Clinical study	Early- and late-onset AD patients	Lactoferrin	Lower lactoferrin levels observed in AD, particularly late-onset disease	Suggests potential utility for disease stratification
Agnello et al., 2024 [16]	Diagnostic accuracy study	AD patients and controls	Aβ, tau-related biomarkers	Automated chemiluminescent assays detected AD-related biomarkers in saliva and plasma	Demonstrates feasibility of standardized laboratory testing

**Table 2:** Main risk factors of Alzheimer’s disease.

Category	Risk Factor	Potential Contribution to Alzheimer’s Disease	References
**Non-modifiable factors**	Advanced age	The strongest risk factor associated with neurodegeneration and cognitive decline	[4,5,17]
	Genetic predisposition (e.g., APOE ε4 allele)	Increased susceptibility to amyloid-β accumulation and AD development	[4,17]
	Female sex	Higher prevalence of AD observed among women, potentially related to hormonal and longevity factors	[4,5]
**Cardiovascular and metabolic factors** [4,17]	Hypertension	Contributes to vascular dysfunction and impaired cerebral perfusion	[4,5,17]
	Diabetes mellitus	Associated with insulin resistance, oxidative stress, and neuroinflammation	[4,5,17]
	Obesity	Promotes chronic systemic inflammation and metabolic dysregulation	[4,5]
	Dyslipidemia	May influence amyloid precursor protein processing and vascular health	[4,17]
**Lifestyle-related factors**	Physical inactivity	Associated with reduced neuroprotection and increased cognitive decline risk	[4,5]
	Unhealthy diet	Contributes to oxidative stress, inflammation, and metabolic disorders	[4,5]
	Smoking	Increases oxidative stress and vascular damage	[4,5]
	Excessive alcohol consumption	Associated with neuronal toxicity and cognitive impairment	[4,5]
**Cognitive and psychosocial factors**	Low educational level	Reduced cognitive reserve may increase susceptibility to dementia	[4,5]
	Social isolation	Associated with accelerated cognitive decline and depression	[4]
	Limited cognitive stimulation	May reduce neuronal plasticity and resilience	[4,5]
**Sleep and mental health factors**	Sleep disturbances	May impair amyloid-β clearance and promote neurodegeneration	[4,17]
	Depression and chronic stress	Associated with neuroinflammation and hippocampal dysfunction	[4,17]
**Inflammatory and systemic factors**	Chronic systemic inflammation	Contributes to neuroinflammatory processes and neuronal damage	[1,17]
	Infections	May trigger immune activation and inflammatory responses	[1,17]
	Periodontitis and oral pathogens	Potential involvement in the oral–brain axis and neuroinflammation	[17,18,19,20,21,22,23]
**Other factors**	Traumatic brain injury	Associated with increased risk of neurodegeneration	[4,5]
	Air pollution exposure	Linked to oxidative stress and neuroinflammatory changes	[4]
	Vitamin deficiencies (e.g., B12, D)	May contribute to cognitive dysfunction and neuronal impairment	[4,5]

**Table 3:** Main salivary biomarkers investigated in Alzheimer’s disease based on the current literature.

Biomarker	Biological Role/Mechanism	Findings in Alzheimer’s Disease	Potential Clinical Relevance	Main Limitations	References
**Amyloid-beta (Aβ42, Aβ40)**	Product of APP cleavage; involved in amyloid plaque formation	Increased salivary Aβ42 levels reported in some AD patients	Potential early diagnostic biomarker reflecting amyloid pathology	Inconsistent results between studies; low standardization	[33,34,35,39,40]
**Tau protein (t-tau, p-tau)**	Microtubule stabilization; hyperphosphorylation leads to NFT formation	Elevated salivary p-tau observed in several studies	May reflect neuronal injury and disease progression	Contradictory findings and limited reproducibility	[11,14,36,37,38,39,40]
**Lactoferrin**	Iron-binding glycoprotein with antimicrobial, anti-inflammatory, and neuroprotective properties	Reduced salivary lactoferrin frequently associated with AD	Potential non-invasive marker for early detection and monitoring	Some studies failed to confirm significant differences	[7,8,9,10,15]
**Exosomes**	Extracellular vesicles carrying proteins, lipids, and nucleic acids	Involved in Aβ aggregation, tau spread, and neuroinflammation	May provide stable and CNS-related biomarker cargo	Isolation and analysis methods remain non-standardized	[41]
**Oxidative stress markers**	Reflect oxidative damage to proteins, lipids, and nucleic acids	Increased oxidative stress reported in AD patients	May indicate neurodegenerative and inflammatory activity	Limited clinical validation and heterogeneous methodologies	[44,45,46,47,48]
**Lipid metabolites**	Participate in APP processing, synaptogenesis, and inflammation	Altered lipid metabolism observed in AD	Potential role in metabolic profiling and disease monitoring	Mostly based on animal and in vitro studies	[42,43,46,48]
**MicroRNAs** **(miRNAs)**	Post-transcriptional regulation of gene expression	Altered salivary miRNA profiles detected in AD	Promising non-invasive molecular biomarkers	Small cohorts and inconsistent biomarker panels	[13,49,50]
**Cell-free DNA/non-coding RNAs**	Reflect cellular injury and inflammatory pathways	Preliminary evidence of altered expression in AD	Potential future biomarker panels	Limited available evidence and lack of standardization	[40,50]
**Inflammatory mediators (cytokines, IL-1β, TNF-α)**	Reflect systemic and local inflammatory responses	Elevated inflammatory markers associated with AD and periodontitis	May support oral–brain axis hypothesis	Low specificity for AD	[1,6,12]

**Table 4:** Hierarchy of evidence and clinical maturity of salivary biomarkers investigated in Alzheimer’s disease.

Biomarker	Level of Evidence	Independent Replication	Potential Clinical Utility
Lactoferrin	High	Yes (partially)	High
Amyloid-β	Moderate	Limited	Moderate
Phosphorylated tau	Moderate	Limited	Moderate
Exosomes	Preliminary	No	Emerging
miRNAs	Preliminary	No	Emerging
Oxidative stress markers	Mechanistic	No	Low
Inflammatory mediators	Mechanistic	No	Low

## Data Availability

No new data were created or analyzed in this study. Data sharing is not applicable in this study.

## Appendix A

**Table A1:** Characteristics of key human studies investigating salivary biomarkers in Alzheimer’s disease.

Study	Study Design	*N* (AD/Control)	Sample Type	Analytical Technique	Biomarker	Main Findings
Carro et al., 2017 [7]	Case–control	274 participants	Whole saliva	ELISA	Lactoferrin	Reduced salivary lactoferrin in MCI and AD
González-Sánchez et al., 2020 [8]	Observational	142 participants	Saliva	ELISA	Lactoferrin	Lower levels associated with amyloid pathology
Gleerup et al., 2021 [9]	Cohort study	222 memory-clinic patients	Saliva	ELISA	Lactoferrin	No diagnostic discrimination
Cui et al., 2022 [11]	Cross-sectional	75 patients with AD and controls	Whole and glandular saliva	ELISA	p-tau	No significant differences observed
Sabaei et al., 2023 [14]	Cross-sectional	70 patients with Early AD and controls	Saliva	ELISA	p-tau	Increased salivary p-tau in AD
Ryu et al., 2023 [13]	Clinical biomarker study	40 AD patients	Salivary exosomes	RT-PCR	miR-485-3p	Associated with brain amyloid deposition
Agnello et al., 2024 [16]	Diagnostic study	42 patients AD and controls	Saliva and plasma	Automated chemiluminescent immunoassay	Aβ, tau	Demonstrated feasibility of automated detection
